# HDAC6 inhibition: a significant potential regulator and therapeutic option to translate into clinical practice in renal transplantation

**DOI:** 10.3389/fimmu.2023.1168848

**Published:** 2023-07-21

**Authors:** Qian-qian Zhang, Wei-jie Zhang, Sheng Chang

**Affiliations:** ^1^ Institute of Organ Transplantation, Tongji Hospital, Tongji Medical College, Huazhong University of Science and Technology, Wuhan, China; ^2^ Key Laboratory of Organ Transplantation, Ministry of Education, NHC Key Laboratory of Organ Transplantation, Key Laboratory of Organ Transplantation, Chinese Academy of Medical Sciences, Wuhan, China

**Keywords:** epigenetics, HDAC6, selective HDAC6 inhibitors, molecular regulation, immunoregulation, renal transplantation, ameliorate outcomes

## Abstract

Histone deacetylase 6 (HDAC6), an almost exclusively cytoplasmic enzyme, plays an essential role in many biological processes and exerts its deacetylation-dependent/independent effects on a variety of target molecules, which has contributed to the flourishing growth of relatively isoform-specific enzyme inhibitors. Renal transplantation (RT) is one of the alternatively preferred treatments and the most cost-effective treatment approaches for the great majority of patients with end-stage renal disease (ESRD). HDAC6 expression and activity have recently been shown to be increased in kidney disease in a number of studies. To date, a substantial amount of validated studies has identified HDAC6 as a pivotal modulator of innate and adaptive immunity, and HDAC6 inhibitors (HDAC6i) are being developed and investigated for use in arrays of immune-related diseases, making HDAC6i a promising therapeutic candidate for the management of a variety of renal diseases. Based on accumulating evidence, HDAC6i markedly open up new avenues for therapeutic intervention to protect against oxidative stress–induced damage, tip the balance in favor of the generation of tolerance-related immune cells, and attenuate fibrosis by inhibiting multiple activations of cell profibrotic signaling pathways. Taken together, we have a point of view that targeting HDAC6 may be a novel approach for the therapeutic strategy of RT-related complications, including consequences of ischemia-reperfusion injury, induction of immune tolerance in transplantation, equilibrium of rejection, and improvement of chronic renal graft interstitial fibrosis after transplantation in patients. Herein, we will elaborate on the unique function of HDAC6, which focuses on therapeutical mechanism of action related to immunological events with a general account of the tantalizing potential to the clinic.

## Graphical Abstract

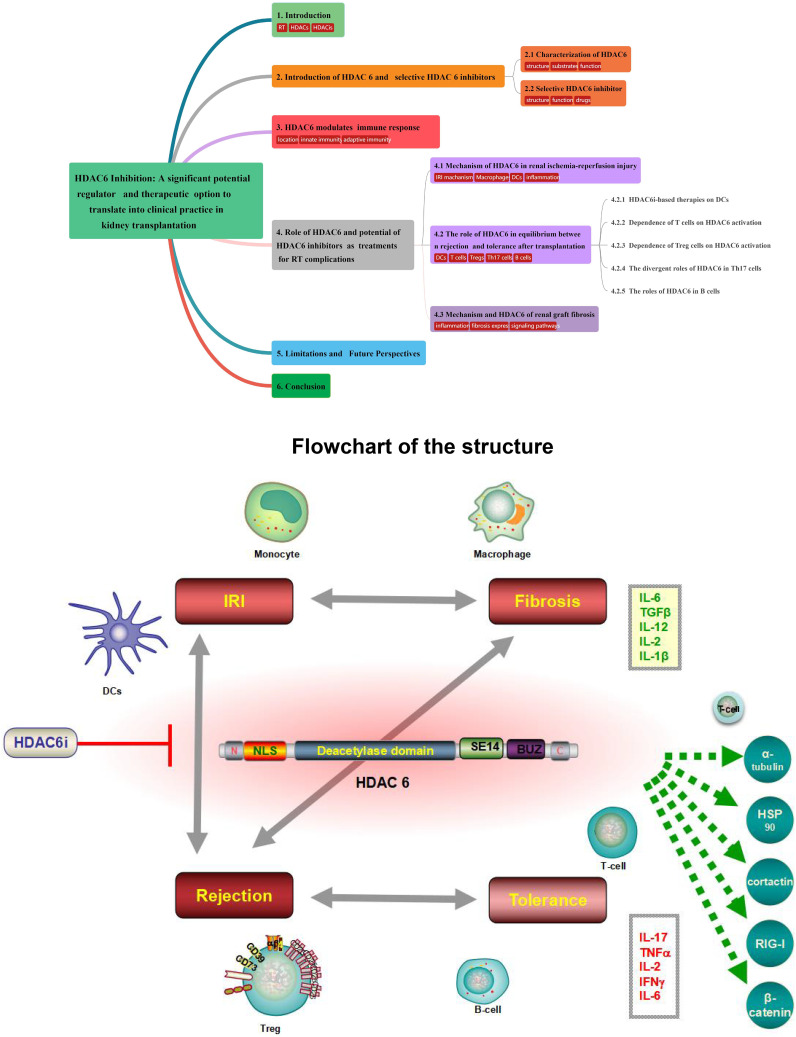

## 1 Introduction

Progressing to end-stage renal disease (ESRD), many chronic kidney disease (CKD) patients require renal replacement therapies (1). The preferred therapy and the most cost-effective treatment approach for the majority of patients with ESRD are actually renal transplantation (RT) (2). Actually, despite ongoing advances in RT, there are still emerging challenges related to immunosuppressive therapy in this field, including ongoing consequences of ischemia reperfusion injury (IRI) (3), ideal goal of immune tolerance (4), equilibrium of rejection (5), and improvement of chronic interstitial fibrosis (6) in patients after transplantation. All these posttransplant conditions are related to the prominent function of the immune system and are responsible for the fate of the renal graft (7). Therefore, optimal management of RT is imperative for improvement of global public health problems. Improving long-term outcomes is now a major focus of RT, with much effort being conducted (8). As advances have been made in both the surgical techniques and immunosuppression (IS) aspects of transplantation, the long-term complications related with RT have been of increasing importance, especially as transplant recipients live longer (9). The induction immunosuppressive regimens currently known to delay the progression of these complications are not effective enough, nor are the triple maintenance regimens (9). Therefore, therapeutic candidates needed to mitigate the development of these clinical dilemmas are under investigation, and histone deacetylase inhibitors (HDACis) may be identified as such important therapeutic applications (10–12).

In eukaryotic cells, histone acetyltransferases (HATs) and histone deacetylases (HDACs) are key enzymes that play an essential role in modifying chromatin structure and thereby modulate gene expression levels, although their possible potential roles in determining cell fate has yet to be fully elucidated (13). They form part of multiprotein complexes that include other proteins known to be involved in activating/repressing transcription, along with antagonistic proteases that play a regulatory role in balancing HAT and HDAC in nucleosomes (14). HATs neutralize the positive charge of histone tails by transferring acetyl groups from acetyl-CoA to the ϵ-NH2 group of lysine residue side-chains, thereby making chromatin looser and promoting gene expression. Nevertheless, deacetylation is associated with gene silencing; to facilitate histone deacetylation and balance the acetylating activities of HAT in chromatin remodeling, HDACs remove acetyl groups from hyperacetylated histones, thereby balancing HATs where the positive charge on the surface of the recombinant lysine increases the binding affinity to the negatively charged surface of DNA (14, 15).

**Figure 1:**
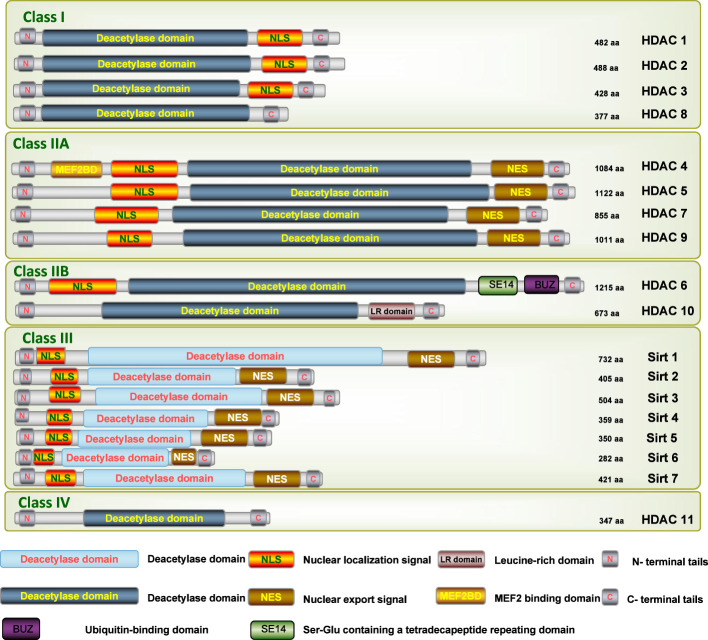
Schematic depiction of the different isoforms of HDAC. N, N-terminus; C, C-terminus; Blue lines depict the length of the protein; Yellow bars depict catalytic domains; Orange bars depict nuclear localization sequences (NLS); Brown bars depict nuclear export sequences (NES1, NES2); Green bar depicts Ser-Glu containing a tetradecapeptide repeating domain (SE14); Purple bar depicts a zinc-finger ubiquitin-binding domain (ZnF-UBD).

As a large family of transcriptional repressors, HDACs play an extremely important role in the epigenetic regulation of a wide range of cellular processes, chromatin structure, gene expression, and physiological functions in various renal diseases (16). Based on homology to yeast HDACs, biological function, and DNA sequence similarity, the family of HDAC isoforms consists of at least 18 characterized members to date, which can be grouped into four classes (**Figure 1**) (17): class I (HDAC1-HDAC3, and HDAC8), class II (HDAC4-HDAC7 and HDAC9-HDAC10), class III (SIRT1-SIRT7), and class IV (HDAC11). Class I, II, and IV HDACs are all Zn_2_+-dependent deacetylases, while class III HDACs are known as nicotinamide adenine dinucleotide (NAD+)-dependent sirtuins (SIRT1-7).

Within the chemical classes of HDAC-inhibiting agents, some HDAC-inhibiting agents act against most HDAC isoforms non-specifically, while others act selectively against a specific HDAC class or isoform (17). Pan HDACis have demonstrated prominent efficacy in treating allergy (18), cancer (19), and inflammatory and autoimmune diseases (20). HDACis have been shown to initiate a remarkable immunosuppressive effect and prolong graft survival in studies evaluating the effect of HDACis on renal injury after RT. Recently, the administered TsA alone doubled the fully the major histocompatability complex (MHC)-mismatched murine cardiac allograft survival carrier, while a subtherapeutic course of rapamycin (RPM) merely extended allograft survival by an additional 2–3 days. Of note, a short course of HDACi therapy was shown to be synergistic with low-dose RPM therapy, promoting permanent cardiac allograft survival and the development of Treg-dependent donor-specific allograft tolerance, as also was the case with islet allografts (21). Regarding mechanism, HDACi therapy increased histone 3 acetylation within the graft, confirming the expected effect of HDACi on protein expression within cardiac allografts, and most of the clear results are associated with increased Foxp3Treg cells within the graft (22).

Nevertheless, their profiles of non-selective HDACi possess a main disadvantage, as it is accompanied by some undesirable effects, including anorexia, nausea, vomiting, diarrhea, thrombocytopenia, neutropenia, anemia, and alteration of serum biochemistry profiles, which severely limit their clinical utility in chronic disease indications (21). In this context, increasing efforts are being made to identify isoform-selective HDAC inhibitors as epigenetic therapeutics (22). A wealth of evidence supported that HDAC subtype–selective inhibitors with fewer adverse effects compared with Pan HDAC inhibitors could be applicable to treat chronic conditions; the evolving trend is the identification of HDAC isoenzyme–selective inhibitors with immunomodulatory function, as well as an elevated safety profile (22). Based on a comprehensive analysis of previous studies, it is more advisable that the HDAC subtype may represent a novel therapeutic target for RT, with many isoform-selective HDAC inhibitors showing positive efficiency (10). Herein, we pay special attention to the role of HDAC6 in the progression of kidney diseases and the tantalizing promise of its inhibitor (23), which could facilitate the advancement of HDAC6-targeted therapeutic methods for complications of RT.

## 2 Introduction of histone deacetylase 6 and selective histone deacetylase 6 inhibitors

### 2.1 Characterization of histone deacetylase 6

Among epigenetic regulation processes of HDACs, HDAC6, a cytoplasmic class IIb HDAC, stands out for its nearly all deacetylation in the cytoplasm and for its catalytic activity involving both dependent and independent mechanisms, which paved an enormously therapeutic potential avenue for identifying its substrates, together with developing highly selective enzyme inhibitors (24). HDAC6 displays important roles in many biological processes and potentially regulates several cellular functions, including cycle regulation, DNA repair, actin-dependent cell motility, and tumor metastasis, respectively (25). Importantly, HDAC6 not only displays a variety of non-histone substrates such as alpha-tubulin (26), heat shock protein 90 (HSP90) (27) and cortactin (28), Ku70 (29), RIG-I (30), p53 (31), STAT3 (32), NF-κB (33), FoxO1 (34), and β-catenin (35), as well as regulate protein degradation, but also plays its mutual effect with several proteins that modulate its deacetylase activity through interactions between proteins.

Uniquely, the HDAC6 isoform displays two functional deacetylase domains in tandem, designated DAC1 and DAC2, respectively, DD1 and DD2, along with a C-terminus hydrolase–like zinc finger domain with conserved regions rich in cysteine and histidine for ubiquitin binding (ZnF-UBP), which is specifically binded to mono- and polyubiquitin chains (**Figure 1**) (36). DD2 is in charge of its deacetylation activity on histones and α-tubulin, according to experimental data using purified HDAC6 (37). Additionally, depending on their acetylation status, HDAC6 shuttle between the cytoplasm and the nucleus, allowing them to interact with miscellaneous cytoplasmic proteins and polyubiquitins, thereby having an influence on cell migration and proliferation, and, together with misfolded proteins, induced cell stress, as well as the processing or degradation of protein aggregates (25).

Meanwhile, the elevated expression and activity of HDAC6 have confirmation of participating in various kidney diseases including acute kidney injury (AKI) (38), polycystic kidney disease (39), lupus nephritis (40), renal cancer (41), and hypertensive nephropathy (42). With enhanced efficacy and less toxicity, selective HDAC6 inhibitors (HDAC6i) are growingly in demand as powerful and promising treatments for multiple pathological manifestations in the kidney including glomerulosclerosis and nephritis, tubulointerstitial inflammation, and renal interstitial fibrosis, which have shown beneficial effects for previous examination using experimental animal models (23).

Collectively, as the specific cytoplasmic localization and the wide range of protein substrates, together with aberrant HDAC6 activity in increased fibrosis (42) and inflammation (43) of kidney, HDAC6 inhibitors have indeed effectively mitigated the progression of immune-related complications in the kidney, indicating that targeting HDAC6 may offer a novel and attractive therapeutic method for complications associated with RT. In this review, we endeavor to have a summary of the role of HDAC6 in biological processes relevant to RT diseases and highlight the effects of HDAC6i and the mechanisms underlying their protection, including IRI, tolerance, rejection, and fibrosis, which would improve recipients’ clinical outcomes and hold great promise as therapeutic agents.

### 2.2 Selective histone deacetylase 6 inhibitor

The majority of hydroxamate-based inhibitors have been shown to be highly effective in inhibiting HDAC6, which are able to form a bidentate complex with zinc ions. The flourishing growth and refinement of crystallization technology have enabled the design of novel HDAC6i with significant therapeutic value. From a structural point of view, HDAC6i are typically composed of three main components: the zinc-binding group (ZBG), which is located at the bottom of the HDAC6 catalytic cavity coordinated with Zn+, by means of an unusual monodentate coordination geometry, or a canonical bidentate Zn_2_+ coordination geometry (44). With the selectivity-determining region, a strong hydrophobic interaction is established by the capping group, “L1 loop pocket,” which occupies the large surface area of the HDAC6 molecule. The linker group binds to HDAC6 *via* hydrogen bonds and bridges between the ZBG and the cap group (44). Moreover, earlier studies have validated that HDAC6 catalytic pocket flexibility allowed protein to undergo structural changes with accommodation of the cap part of the ligands under investigation (45). Currently, the most widely investigated inhibitors of selective small molecule HDAC6 mainly include Rocilinostat (ACY-1215), Citarinostat (ACY-241), Tubacin, CKD-506, Tubastatin A(TA), BML-281, and LTB2 (46). The most successful anti-tumor HDAC6 inhibitors to date are ACY-1215 (47) and ACY-241 (48), which are oral selective HDAC6i under clinical investigation for the treatment of refractory multiple myeloma (MM). Subsequently, a series of carbazole hydroxamic acids with alkyl and alkylaryl linker groups were synthesized by Butler et al. in accordance with a wider and shallower channel of HDAC6. Meanwhile, with an IC50 of 15 nM against HDAC6, TA was found to have anti-HDAC6 function (49). A potent and selective inhibitor of the HDAC6 enzyme is hydroxamic acid (M808), with improved selectivity and solubility over the structural analog ACY-1215 (50). **Table 1** summarizes some representative HDAC6i, together with their inhibitory functions, currently in clinical trials (disease), reported related diseases, the pathomechanisms of complication therapy of RT-related complications.

**Table 1:** A summary of representative HDAC6 is 1–21 along with their inhibitory activities, original targets (disease), mechanisms of therapy for RT related complications and respective limitations.

No./Name	Structure	IC50	Currenty in Clinical Trials (Disease)	Reported related diseases	Mechanism of therapy for RT related complications
1 Tubacin	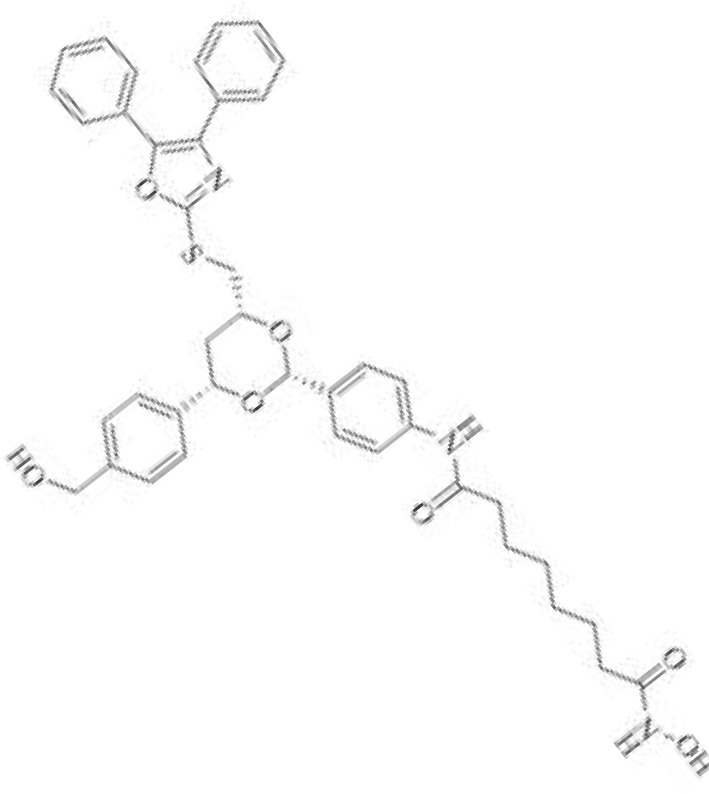	4nM	HDAC6 (Anticancer) (51)	1. Polycystic kidney disease (39).	1. Amelioration of learning and memory deficits.
2 TA	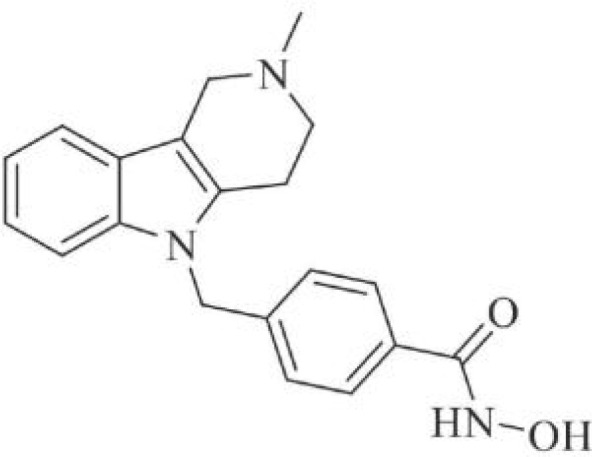	15 nM	HDAC6 (NDs) (49)	1. Rhabdomyolysis-induced nephropathy (52) 2. Ischemia- and Cisplatin-Induced AKI (53)	2. Inhibit the mitosis of Teffs.
3 MPT0G2	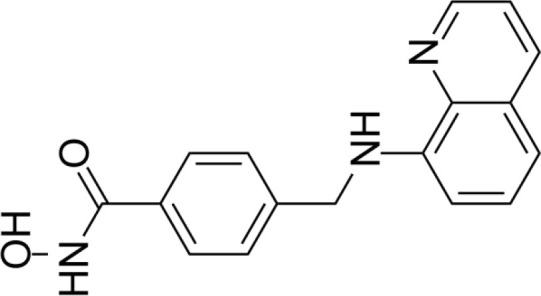	0.291 μM	HDAC6 (Alzheimer's disease, AD) (54)	Not reported so far	Not reported so far.
4 ACY-7	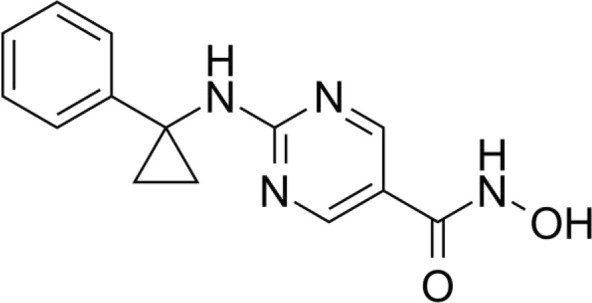	1.7nM	HDAC6 (Multiple myeloma, MM) (55)	1. Polycystic kidney disease (56) 2. SLE (57)	1. Attenuate IL-1β production. 2. Increased Tregs. 3. Decrease pro-B cell differentiation, Bcl-2:Bax ratio. 4. Suppression of T cells.
5 ACY- 1215,Ricolino stat	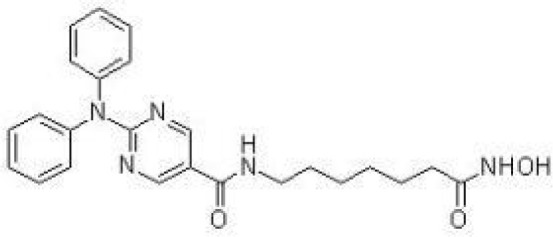	4.7nM	HDAC6 (MM) (47)	1. UUO-induced fibrotic kidneys (58) 2. Polycystic kidney disease (59)	1. Suppress Th2 cytokines. 2. Reduce differentiation of Th17 cells. 3. Modulate CD8 T-cell activation and functions. 4. Decrease M1 and cytokines.
6 CKD-5	Not discover	Not discovered.	HDAC6 (Rheumatoid Arthritis, RA) (60)	1. Systemic lupus erythematosus (SLE) (61)	1. Decrease levels of IFN-γ, IL- 1β, IL-4, IL-6, IP-10, MCP-1, and CCL4 in the kidney extracts of mice with lupus nephritis 2. Repress innate immune systems such as production of TNF-α and type1 interferons 3. RegulatE T cell- and macrophage-mediated peripheral immune responses. 4. Downregulate the expression of IFN-γ and IL-17A. 5. Downregulate the levels of pro-inflammatory cytokines
7 Nexturastat A (NexA	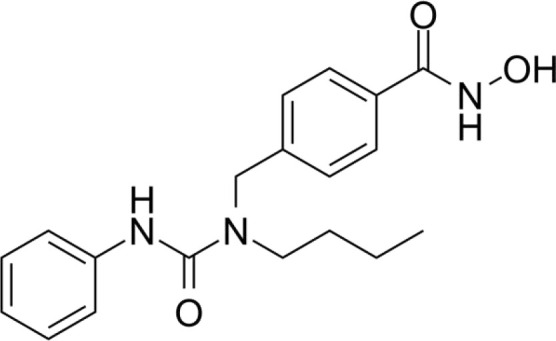	5.02nM	HDAC6 (MM) (62)	Not reported so far.	1. Suppresse the mRNA levels of pro-inflammatory cytokines IL-1β and IL-6 2. Decrease the anti-inflammatory phenotype of macrophages and down-regulation of immunosuppressive proteins
8 Citarinostat (ACY-241)	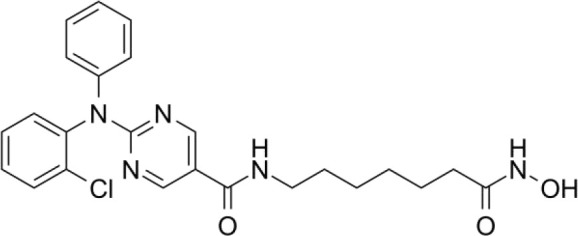	2.6 nM	HDAC6 (MM) (63)	Not reported so far.	Not reported so far.
9 Benzamide (HPOB)	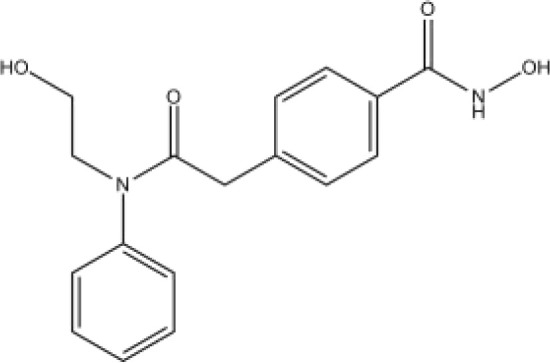	56 nM	HDAC6 (Antitumor) (64)	Not reported so far.	3. Not reported so far.
10 BML-281 CAY10603	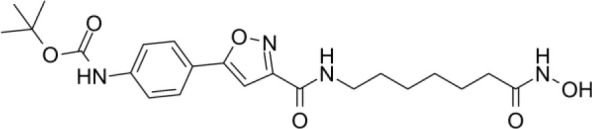	0.002 nM	HDAC6 1. (non-small cell lung cancer, NSCLC)) (65)	Not reported so far.	Not reported so far.

## 3 Histone deacetylase 6 modulates immune response

The immune system falls into two general types, which are innate and adaptive (66). Neutrophils, macrophages, dendritic cells (DCs), natural killer cells, gamma and gamma delta cells and other innate lymphoid cells, and complement system members, and mediators of inflammation, are among the different cell types involved in the innate immune system (67), which acts on most immunological events following RT with an important role and recognize immune antigens that are fast and lasting through pattern-recognition receptorsin a very short time frame (68). Through migration and maturation in the kidney, DCs are involved in inducing and regulating innate and adaptive immunity (69). Generally considered to be antigen-presenting cells (APCs) and a core participant for all immune responses, DCs are not only particularly effective immunogens in inflammation but also critical for inducing and maintaining self-tolerance in the context of stable immune status (69). Furthermore, selective HDAC6i is also necessary for the recovery of innate immune cells in the bone marrow, followed by reducing stress and immune atrophy and apoptosis (70). Macrophages, as the key effector modulators of the innate immune system, play a predominant role in inflammatory initiation, resolution, tissue repair, and regeneration (71). Recent work has presented that monocytes and macrophages directly recognize allorecognition (72), which is independent of lymphoid cells and induces the maturation of APC; thus, the initiation and maintenance of an adaptive allogeneic immune response is possible.

Rapid response of innate immune cells develop an immunological memory, whereas a delayed response is seen in adaptive immune cells and can be days away from full development (73). One of the most vigorous elements of the immune system is the adaptive immunity system, where the main immunoreactions are generated by T and B cells, whose products are of crucial importance for immune protection against pathogens and inflammation (67). In the process of T-cell receptor (TCR) recognition of Th cells, CD4 is the receptor and participator of the Th cell TCR for antigen recognition. CD8, a leukocyte differentiation antigen, is used to assist the TCR to recognize antigens and take part in the signal transduction of T-cell activation. Based on the subsets, B cells play a diverse role toward organ allografts, either enhancing or suppressing immunity (67). What is interesting is that the communication between the innate and adaptive immune responses during RT is bidirectional, as it is now clear to see that activating the adaptive immune response can be the cause of kidney damage induced by cellular and molecular components of innate immunity (74).

Relying on the cytoskeleton to undergo constant reorganization, including acetylation or deacetylation, HDAC6 influences a wide range of cellular functions and cytoplasm shape, such as cell signaling, activation, survival, motility, and protein degradation (24, 25, 75), which ultimately has an impact on cell function. Various studies have since shown that HDAC6 activity regulation, which is expressed in both the nucleus and the cytoplasm, have identified a significantly critical role for the behavior in innate and adaptive immunity (76), together with modulating the inflammatory genes (24, 28). In clinical RT, activation of the innate immune response can be triggered by certain elements (77). Brain death itself may accelerate the systemic production and release of proinflammatory cytokines (monocyte chemotactic peptide-1 and interleukin-6) in donation after brain death (DBD), contributing to activating innate immune pathways, such as recruiting and activating monocytes in multiple organs, including the kidneys (78). Based on previous and current studies, HDAC6i conduct suppression of excessive inflammation by inhibiting DCs, with prevention of damage to kidney function. Through enabling macrophages to the selective recognition and elimination of allogeneic targets, HDAC6 deficiency or HDACi also produces a protective immune response. HDAC6 therefore has the critical role in regulation of innate immune responses, and HDAC6 inhibition can sustain the ability to ameliorate the inflammatory response at a later stage. A previous study reported that HDAC6 affects the activation of antigen-dependent CD4 T cells (67), and another revealed that HDAC6 plays a role in CD8 T-cell cytotoxic function (79). In addition to suppressing B- and T-cell activation, HDAC6i also attenuated T-cell differentiation into several other subtypes known to be regulated in immune-related diseases, both *in vivo* and *in vitro* (80). There were pieces of evidence showing that abnormal B-cell differentiation was associated with a less severe form of kidney disease, which could be corrected by selective HDAC6 inhibition (81).

Inhibition of HDAC6 therefore has pleiotropic effects on the immune system. In the future, for current clinical relevance, therapeutics targeting HDAC6 may have particular potential for the early intervention strategy of RT-related diseases. Beyond that, the effects of later intervention with HDAC6 inhibition, starting after the onset of RT-related disease, are more important to investigate. These are discussed in detail in the following sections.

## 4 Role of histone deacetylase 6 and potential of histone deacetylase 6 inhibitors as treatments for RT complications

### 4.1 Mechanism of histone deacetylase 6 in renal IRI

**Figure 2:**
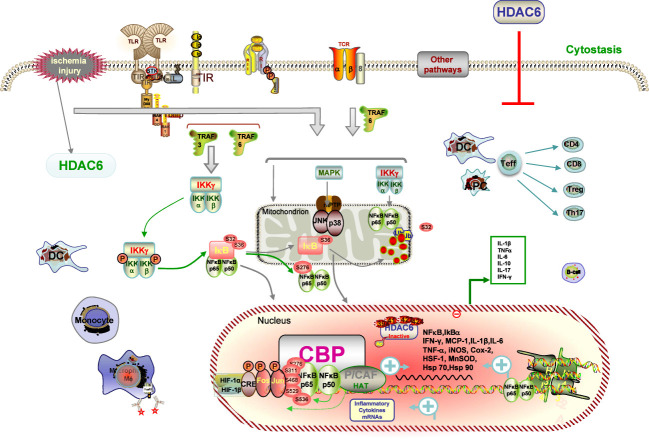
Description of HDAC6 on IRI pathways. IRI causes immune cells of both the innate and adaptive immune systems, such as neutrophils, DCs, macrophages, and lymphocyte subsets contribute to the pathogenesis and pathological processes of IRI. IRI-generated ROS promotes activation of the NF-κB and AP-1 signaling pathways with subsequent transcription and production of inflammatory cytokines signaling mechanisms and transcriptional regulatory pathways. HDAC6 deacetylate histone and non-histone proteins, which blockage-elicited renal protection is associated with inhibition of suppression of inflammatory responses, and reduction of oxidative stress. HDAC6 inhibitions dampen the expression of ROS with subsequent transcription and production of proinflammatory cytokines by inhibiting the NF-κB and AP-1 signaling pathways.

IRI, a common posttransplant event, increases the risk of delayed graft function (DGF), setting the stage for graft rejection and long-term graft loss (3). Accumulating evidence indicates that early warning signs of danger linked to IRI spell innate immune activation, together with promoting graft rejection and inhibiting tolerance occurrence (78, 82). Innate and adaptive immune cells such as neutrophils, including DCs, macrophages, and lymphocyte subsets, have been shown to be involved in the pathological processes of IRI, resulting in the release of potent cytokines and chemokines, not least certain subpopulations involved in the repair process (82, 83). Despite decades of research into the mechanism of IRI, no clinically approved maneuvers to mitigate this process have been the mainstay of clinical practice for the modification of the negative influence of IRI associated with both short- and long-term outcomes of renal transplant recipients. As such, we focused on the critical role of HDAC6 in IRI, with the aim of identifying potential targets for preventive therapeutics (**Figure 2**).

Also, it is now evident that HDAC6 deacetylates non-histone proteins to take part in broader signaling and transcriptional pathways (27–35). These targets including Hsp70, Hsp90, and heat shock factor protein-1 (HSF-1), known elements and highly expressed during IRI, were acetylated during stress situation, leading to the nuclear translocation and induction of several genes (84, 85). Numerous studies have demonstrated that HDAC6 inhibition is protective in preclinical models of myocardial infarction (86) and IRI (53). Furthermore, HDAC6i have also been the investigated subject in some acute injury models and have presented a positive protective effect. For example, TA improved long-term survival in a lethal sepsis model (86) and ameliorated stroke-related brain infarction and functional deficits (87). It has already been demonstrated that inhibition of HDAC6 can attenuate kidney injury in a 5/6 nephrectomy model of CKD and in rhabdomyolysis- or cisplatin-induced models of AKI (52).

There are several mechanisms by which HDAC6 inhibition may promote a good protective effect during renal IRI. Among these, more obvious data indicated that HDAC6 is a crucial regulator of macrophages (88). There is increasing evidence that macrophages are of paramount importance in the development and progression of renal inflammation and may be mediators of inflammation through surface-expressed receptors (71). Upon activation, macrophages release proteolytic enzymes and inflammatory cytokines such as TNF-α, TGF-β, IL-1β, and IFN-γ (71). After IRI, monocytes infiltrate the renal interstitial space and differentiate into proinflammatory macrophages, known as M1, in response to the initial kidney injury, and subsequently switch to a form called M2, which promotes renal repair (71, 72, 89). Overexpression of HDAC6 results in a spontaneous proinflammatory macrophage response, whereas HDAC6 deficiency has been reported to suppress the immune response during inflammation by ameliorating the monocyte-derived macrophage infiltration and a heavy influx of circulating monocytes (71). Stimulation of macrophages with lipopolysaccharide results in translocation of HDAC6 and cortactin from the cytosol to the periphery, with promotion of filopodial protrusion and enhancement of microtubule acetylation around the microtubule hub, all of which are abolished by HDAC6 deficiency or HDAC6i. Previous studies have mentioned that inhibition of HDAC6 by TA and M808 gave rise to effective alleviation of expression of multiple cytokines and chemokines, as well as elevated macrophage migration and infiltration into the injured kidney in a murine rheumatoid arthritis (RA) model and a monocyte (monocytic cell line U937) migration assay (43, 52). An experimental study confirmed that TA treatment significantly hampered the production of proinflammatory cytokines like MCP-1, TNF-α, and IL-6 by monocytes in rhabdomyolysis-induced AKI (52, 53). Moreover, provided further corroborating evidence, another study supported that TA remarkably enhanced the phagocytic capacity of RAW264.7 macrophages (90). In conclusion, these findings implicate that HDAC6i may represent a potent target for the treatment of macrophage-associated immune diseases with great promise.

DCs can facilitate harmful immune activations, but they are also responsible for beneficial functions in protecting kidney from IRI (83). Serving as a “bridge” between the innate immune system and adaptive immune systems, DCs are a section of the inflammatory response following IRI in the kidney (74, 91). After exposure to an antigen during IRI, DCs migrate to the damaged tissue where they expose antigens to adaptive immune cells (83). Current studies tend to suggest that HIF-1α triggers the maturation of DCs and subsequently damages renal function (92). Furthermore, DCs are thought to have important implications in the early pathophysiology of IRI development within the first 24 h after IRI (74). During the post-IRI period, intra-renal DCs can activate NKT cells and amplify the innate immune response (83). All DC subsets interact with the various effector T cells (Teff), including CD4 T cells, CD8 T cells, and Tregs, induced by IRI procedures, although the classification remains controversial, according to different effects (69). The presence of the DC population was known to be reduced to basal levels in the NextA combination arms from a previous study (93). Recently, it has been suggested that TNF-α, which is also secreted by chlamydia-infected DCs, induces HDAC6 (94). In agreement with these observations, LPS was also found to strongly upregulate the expression of HDAC6 in APCs. On the contrary, HDAC6 inhibition has an effect on the downstream effects of TNF-α. Furthermore, the therapeutic regimen of DCs with selective HDAC6i CKD-506 mitigated the progression of T-cell activation *via* diminishing the production and release of an amount of immunosuppressive cytokine, IL-10, on the basis of the IL-10 colitis model (95).

As described earlier, the renal protection elicited by HDAC6 blockage is associated with the restraint of inflammation and the decreased oxidative stress (96). A previous study investigated that blocking HDAC6 with TA could interfere with oxidative stress triggered in the mouse model of rhabdomyolysis-induced AKI, which effectively lessens malondialdehyde (MDA) levels, preserves the expression of superoxide dismutase (SOD), two representative biomarkers of oxidative stress in the kidney (52). The production of Reactive oxygen species (ROS) and release of cytokines are all cellular events released during reperfusion that result in the renal injury from donor and subsequent generation of DGF (3, 82). Overexpression of HDAC6 during IRI upregulates the activity of Nicotinamide Adenine Dinucleotide Phosphate (NADPH) oxidase with ascending generation of ROS (97). The generated ROS fuels the activation of the NF-κB and AP-1 mechanism pathways and then transcribed and produced proinflammatory cytokines (98). Several reports have reported that HDAC6 inhibition with TA dampens the expression of ROS with subsequent transcription of the NF-κB and AP-1 mechanism pathways, conducted *in vitro* and *in vivo* studies, including various chronic murine colitis with the IL-10 KO mouse and adoptive transfer models (99). By upregulating the expression and activity of NADPH oxidase, studies of HDAC6 overexpression in macrophages significantly triggering the generation of an inflammatory cascade and ACY-1215 decreased the inflammatory response in LPS-induced RAW264.7 macrophages (100). Another preliminary work has shown that HDAC6 participated in genotoxic stress induced by DNA damage agents (101). Taken together, HDAC6i markedly protects against oxidative stress–induced damage, which is of particular importance for immune cells exposed to high ROS concentrations (99, 102). All these findings lend strong support to the fact that HDAC6i represents a therapeutic potential for treating renal IRI.

### 4.2 The role of histone deacetylase 6 in equilibrium between rejection and tolerance after transplantation

**Figure 3:**
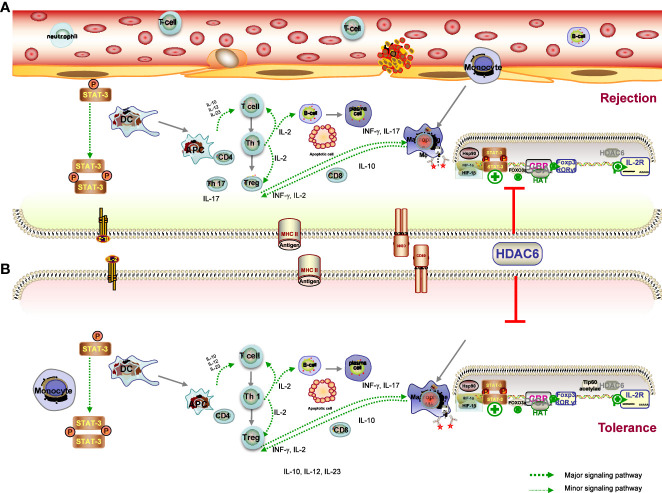
Mechanisms of graft tolerance **(A)** and rejection **(B)** consist of complex processes involving the induction of proinflammatory APCs and tolerogenic DCs, leading to a different microenvironment. (1) HDAC6 promotes the expression of T-bet in the Th1 master regulating the ratio of Th1 to Th2, which also controls the population of CD4 cells, and the CD4T-bet/CD4T-GATA-3 ratio was significantly reduced in the HDAC6i group. in the HDAC6i group. (2) Acetylation promotes Foxp3 dimerisation, DNA binding and transcriptional activity, and expansion of the Treg population, and is ultimately associated with immune tolerance. The effects are likely to be related to increased Foxp3 acetylation and consequent expansion of Tregs. (3) HDAC6 inhibitor upregulates the Treg cell population through the STAT3/IL-10 tolerogenic axis to overcome immune tolerance and tilt the balance towards T-cell immunity. HDAC6 improved the expression levels of TF Rora in Th17, IL-17A secretion in Th17 cells and IL-4 levels secreted by Tregs, Th2 induced, as well as the differentiation of TH17/Treg imbalance. HDAC6i has therapeutic effects on the pathology of acute rejection by targeting the HDAC6/HIF-1a/ROR gt/Th17 signalling axis.

The ultimate goal of transplantation is to achieve donor-specific tolerance, where recipients would enjoy long-term graft survival without the need for lifelong immunosuppressive drugs (103). Despite undeniable advances in RT techniques and IS, immunological rejection is the major cause of graft dysfunction and ultimately graft loss (104). There is compelling evidence that common immunosuppressants have no significant effect on long-term outcome of solid organ transplantation (SOT) because the prevention of chronic graft rejection is less effective with these drugs. Meanwhile, transplant recipients are at significant risk of side effects, infection, and malignancy due to lifelong systemic immunosuppression (105). Many efforts have been made to achieve graft tolerance, and researchers have attempted numerous approaches to induce immune tolerance toward the donor graft in transplant recipients (4, 106). Given that both rejection and tolerance are influenced by the innate and adaptive immune systems, the ideal strategy would be to regulate B cells, DCs, and macrophages in a concerted manner, rather than targeting T cells alone, all of which lead to both the acute and chronic rejection response (107). This leads to the following result: understanding how the immune system works in tolerance and rejection of transplanted organs is of vital importance for the development of the improved therapeutics and better results for the recipient. HDAC6 has emerged as a hopeful policy in recent times to modulate antidonor immune reactions, allowing minimization of immunosuppressive drugs and induction of tolerance (50, 108). The hypothesis is supported by several lines of evidence that HDAC6 deacetylase activity is emerging as a key regulator of the immune response, the targeted inhibition of which has the potential to reduce the risk of organ rejection after transplantation (23, 40, 43). In this chapter, we describe the role of HDAC6 in the many immune cells and different mechanisms participating in the rejection (**Figure 3A**) and tolerance (**Figure 3B**) and discuss the popular and promising new HDAC6i approaches employed to treat transplant recipients.

#### 4.2.1 Histone deacetylase 6 inhibitor–based therapies on dendritic cells

DCs are potent APCs that play important and diverse roles in initiating and regulating antigen-specific immune responses by capturing, processing, and presenting antigens (69). Acute and chronic allograft rejection is induced by DC–lymphocyte interaction with the assistance of other immune cells (109). During the process of graft migration and maturation to induce and regulate adaptive immunity, DCs can adopt different phenotypes—rejection-related DCs cause acute or chronic graft injury, whereas tolerogenic DCs help to prevent kidney damage by suppressing the overwhelming immune response. Through the production of cytokines (including IL-10, IL-12, and IL-23), DCs also play an essential role in the modulation of innate and adaptive immunity (69, 109). Of note, HDAC6 of specific APC genes plays a critical determinant of the chromatin substrate, which has been shown to be involved in immune synapse formation and immune response regulation (94). Disruption of HDAC6 leads to the induction of proinflammatory APCs (secreting the proinflammatory cytokines IFNγ, IL-6, IL-1β, IL-12, and IL-23) and tolerogenic DCs (secreting the anti-inflammatory cytokine IL-10), which are critical for T-cell activation and induction of tolerance, respectively (94). The heterogeneity of the DCs and the states of their activation provides researchers with a greater range of options to define and manipulate the immune response researchers with a greater range of options to define and manipulate the immune response through these specialized leukocytes (110). The cytokine IL-10 and its immunosuppressive effects played a more important role in the generation of tolerant APCs. Expanding on these properties, Chromatin Immunoprecipitation (ChIP) analysis has also presented that HDAC6 regulates STAT3/IL-10 signaling activity, leading to increased antigen presentation (94). Together, previous work presented that HDAC6 activity is of paramount importance in the immune system by regulating DC-derived cytokines, which are of paramount importance in the modulation of the polarization of T cells, the activation of NK cells, and inflammation. HDAC6 thus opens up new avenues for therapeutic intervention to tip the balance in favor of the generation of APCs that are able to induce both up- and downregulation of important surface molecules.

#### 4.2.2 Dependence of T cells on histone deacetylase 6 activation

Directional regulation of Th cell subset differentiation and induction of immune deviation is beneficial for inhibition of graft rejection and the induction of transplant tolerance (111). In the periphery, the goal of tolerance is to inhibit mature alloreactive T and B cells through mechanisms such as deletion, anergy, and regulation (112). HDAC6 is paramount to regulate the immune system, among other functions. In a murine model of cecal ligation and puncture (CLP), TA has been proven to alter the blood cell composition, along with lymphocyte population restoration (84). Previous studies have also shown that CKD-506 directly suppressed the proliferation of pathogenic lymphocytes (88). As clearly demonstrated in an immunization study, overexpression of HDAC6 contributes to increased T-cell migration and chemotactic capacity (113), whereas HDAC6 deficiency results in impaired CD8 T-cell functions. It has been demonstrated that the hydroxamic acid HDAC6 inhibitor ACY-1215 acts directly on T cells to inhibit antigen-specific CD8 T-cell differentiation and proliferation during skin inflammation (114). It is important to note that the development and function of CD4 and CD8 T cells were invested to be normal in CD4-specific Hdac6 knockout mice (80). In addition, qRT-PCR results showed that ACY-1215 also suppressed the upregulation of IL-2 and IFN-γ expression induced by CD8 T cells with anti-CD3/CD28 antibody stimulation in the Contact hypersensitivity (CHS) model (115). The results also presented that the CD4 T-cell proliferation and cytokine production were decreased (115). As mentioned in a previous work, phenotypic analyses of the effector T-cell (Teff) indicated an increased apoptosis of T-acute lymphoblastic leukemia (ALL) cells, following reduced Notch3 expression in the HDAC6 silencing group by specific short hairpin ribonucleic acid vector (shRNA) (116).

Hence, a regulation in T-bet expression of the Th1 master regulator and the ratio of Th1 to Th2 in the spleen may have resulted in the enhancement in rejected graft presentations (104). Analysis of the master regulator of Th1 and Th2 by CD4 cells indicated that the CKD506 group was less prone to express the Th1 differentiation factor T-bet and showed decreased levels of the Th1 cytokine IL-2 in a mouse model of systemic lupus erythematosus (61). In addition, the Th1/Th2 ratio, which controls CD4 cells, and the CD4T-bet/CD4T-GATA-3 ratio were significantly reduced in the CKD506 group compared to the vehicle group (61). It is known from previous work that T-cell migration and chemotaxis were increased in T-cell overexpression of HDAC6 (113). Another finding suggested that CKD-506 attenuated the clinical manifestations of EAE in mice by suppressing T-cell infiltration into the spinal cord and reducing the levels of Th1 cell–related inflammatory cytokines in the peripheral blood and spinal cord of mice (117). The evidence upon all data leads to a just conclusion that HDAC6 might make absolute sense in the activation of Teff cells and in their functions and that HDAC6i might be favorable in the treatment of Teff cell–mediated diseases, including rejection.

#### 4.2.3 Dependence of Treg cells on histone deacetylase 6 activation

Tregs are active participants in the regulation of immune system homeostasis, and alteration of their phenotype is involved in the process of tolerance induction by suppressing naive and subsequent T cell responses, producing TGF-β or inhibiting Teff cells, thereby preventing allograft rejection (118, 119). The pharmacological function of Treg suppression in pathological conditions is an increasingly vibrant area of research. How Tregs therapy is used in SOT is specifically related to the goal of achieving tolerance, along with the reduction or elimination of immunosuppressants, as well as tissue repair and resistance to rejection (120, 121). On another level, established autoimmune diseases can be reversed or even cured by Tregs therapies, which may be effective in controlling autoimmune diseases in animal models (118). Nevertheless, a crucial issue related to Treg therapy is to ensure that the administration of Tregs is precarious and that there may be a decline in the suppressive effect in the body over time (120). Increased Treg proliferation and function exerted by selective HDAC6 inhibitors probably an extremely reasonable regimen for the therapeutic strategy of the organ transplantation, particularly in tolerance.

A previous study reported that Foxp3 transcription factor, a key transcription factor involved in Treg development and activity, was deacetylated under the regulation of HDAC6, and HDAC6 knockout or HDAC6i enhanced the suppressive effect of Treg (122, 123). Previous reports indicate that HDAC6 was presented at a level several times higher in the Tregs than in the Tcon cells, and HDAC6−knockout mice are immunocompetent and inclined to be greater *in vitro*/*vivo* suppression than WT Tregs (124). Another considerable phenomenon associated with transplant models is that a strong determinant of Treg-dependent resistance is the presence or absence of HDAC6 within Tregs to allograft rejection, underscoring the significance of HDAC6 as a treatment strategy for Treg modulation (108). Preliminary work by Yang J et al. presents that there are Hdac6 KO Treg downregulate five MHC class II molecules, manifesting that HDAC6 could help the expression of MHC class II molecules among Treg (125). The published study reasonably concluded that the HDAC6-specific inhibitor promotes the suppressive activity of Treg in the model of fully MHC incompatible skin allograft rejection (108). In a recent study of a cardiac allograft model, the presence or absence of the HDAC6 molecule alone could determine acute rejection or long-term engraftment (121). In addition, long-term graft survival was also observed in HDAC6-/- mice, but not in WT mice, when recipients in fully immunocompetent hosts received a subtherapeutic dose of rapamycin (126).

Beier and colleagues provided evidence to support the previous study that HDAC6 is involved in promoting other transcription factor activities necessary for Treg development and activation, such as cyclic adenosine monophosphate response element-binding protein (125). More evidence, furthermore, suggested that HDAC6 deficiency took part in the stabilizing the acetylated form of IL-2, which mediates STAT5, thereby promoting its transcriptional activity (125). In terms of other molecular mechanisms, previous studies have confirmed that Foxp3 complexes with HSP70, with increased HSP70 promoting Treg activity and efficacy under stress conditions, and subsequent inhibition of HSP70 impairing Treg activity and efficacy (127). Interestingly, there is strong evidence that HDAC6 or HSP90 inhibitors produce similar results in models of inflammation and autoimmunity, that is, HDAC6i or HSP90i can rescue Tregs population and HSP90-dependent enhancement of Treg function with HDAC6i (128). Taken together, HDAC6 is an important regulator of Treg plasticity that determines the fate of plastic Tregs, which promotes expression of Th subset regulators in Tregs and regulates plasticity and heterogeneity of Tregs. Enhancement of the suppressive function of Tregs with HDAC6i is an extremely potential target for the achievement of tolerance as well as for helping prevent graft rejection.

#### 4.2.4 The divergent roles of histone deacetylase 6 in Th17 cells

There is accumulating evidence to suggest that Th1 cells result in the progress of both acute and chronic rejection of the allograft following transplantation of a variety of organs, including the kidney (129). Th17 cells, a subset of T lymphocytes, are known to be involved in the pathogenesis of many autoimmune diseases through the production of proinflammatory cytokines such as IL-17, which is known to regulate many inflammatory diseases (130, 131). Preliminary findings have presented that Th17 cells and their secretion of IL-17 are involved not solely in the course of acute rejection but also in the development of iTreg differentiation–associated tolerance induction (132). Interestingly, accumulating evidence exhibits that the ultimate development of TH17 has a closely reciprocal relationship with Tregs fate and arises from a common T-cell precursor (133). Additionally, a study of systemic autoimmune disease suggested that the transfer of Tregs enhanced IL-17 production in *in vivo* (133). There is a range of evidence that the favorable approach of HDAC6i inhibits the activity of Th17 cells in lung allografts and ultimately presents a protective effect (134). However, other results have shown that HDAC6 improved the expression levels of TF Tbx21 in Th1, together with expression of TF Rora in Th17 and IL-4 levels secreted by Tregs induced by Th2 (133).

Available data confirm that differentiated Tregs, probably through their TGF-β production, contribute to the promotion of the Th17 cell differentiation fate of CD4 T cells, with IL-6 levels determining the transcriptional balance between Foxp3 and RORγt in the presence of TGF-β (135). A previous study showed that Th17 and IL-17+ γδ T cells were depleted in recipients following adoptive transfer of induced Treg cells (iTregs) and attenuated the pathological manifestation of acute rejection by suppressing Th17 cell accumulation in lung allograft recipients (132, 135). Previous data demonstrated that ACY-1215 decreased IL-17A secretion in Th17 cell culture system, as well as the Th17 cell differentiation, which was confirmed in the experimental autoimmune thyroiditis (EAT) mouse model (134). Also, a recent study exhibited that the HDAC6-specific inhibitor TA showed the same result in an orthotopic mouse lung transplantation model, demonstrating the protective role of HDAC6 in lung allografts through the suppression of Th17 cells (136). It is presented that TA suppressed the differentiation and function of Th17 cells and ultimately improved the attenuation of acute rejection of lung allografts and prolonged survival (136). Furthermore, a dramatically increased rate of Tregs to CD4 T cells in both allograft and spleen isolation in the TA-treated group is also noted, as well as an obviously increased IL-10 level of Tregs in the TA-treated group in the animal lung transplantation experiment. From another perspective, HDAC6 depletion restored the suppressive activity of Tregs in models of autoimmunity and inflammation (136). The above results correlate well with previous reports showing that HDAC6 inhibition/deficiency enhances Treg function and inhibits TH17 lineage commitment, thereby readjusting Th17 and Treg cell fractions. In allograft recipients, all reliable evidence suggests that HDAC6i therapy had a general and substantial effect on the fractions of Th17 and Tregs.

Other mechanistic studies have mainly involved HIF, the role of an essential regulator in T-cell fate determination. It has been reported that HIF-1, the transcription factor in response to hypoxia (137), actually regulates the balance of TH17/Treg (138). It has been demonstrated that high HIF-1α expression was observed in lung allograft transplantation and that HIF-1α is required for Th17 cell development (136). HIF-1α KO mice were protected from acute rejection in the lung allograft and manifested an apparently reduced infiltration of Th17 cells. Consistent with previous presentations, high HIF-1α expression of T cells can be induced by hypoxic and normoxic conditions (137). A previous study has identified an important target through which HIF-1α regulates Th17 cell expansion and function, such as the HIF-1α-dependent RORγt pathway, as reduced RORγt expression was observed in HIF-1α-deficient mice (139). Other studies have reported that decreased RORγt levels are of greatest value for HIF-1α, resulting in downregulation of Th17 cells and attenuated acute rejection of lung allografts (132). A recent study demonstrated that TA downregulated HIF-1α transcriptional activity of nucleus pulposus cells and the protein expression level, and in the TA-treated lung transplant group, reduced HIF-1α expression may be in relation to the improvement of inflammation responses (140). Collectively, there is significant evidence that HDAC6i, TA, has therapeutic effects on the pathological lesions of acute rejection in the lung allograft models by targeting the HDAC6/HIF-1α/ROR γt/Th17-signaling axis. Given the above evidence of plasticity between TH17 and Treg programs, attention to manipulating the balance of these cell lineage decisions may suggest novel strategies for treating RT fields associated with TH17/Treg imbalance.

#### 4.2.5 The roles of histone deacetylase 6 in B cells

Playing an essential role in the immunological response, B cells can promote rejection through the production of antibodies, proinflammatory cytokine expression, and presenting antigens to T-cells, which leads to the activation and differentiation of T cells (105). B cells also evolve into antibody-secreting cells in which the antibodies act as opsonins to facilitate activation of DCs and T-cell responses, which can also produce IL-10 and TGF-β to induce Treg differentiation, thereby reducing the severity of inflammatory diseases (140, 141). It has long been known that the expression level in B cells and splenic B cells was significantly increased in diseased MRL/MpJ-Faslpr (MRL/lpr) mice (84, 142). It was previously reported that pre-B cells treated with ACY-738 were able to suppress pre-B cell proliferation by increasing Bax expression and reducing the Bcl-2:Bax ratio, thereby supporting a proapoptotic environment *in vitro* (143). Furthermore, ACY-738 treatment restored early pre-B-cell proportions but reduced late pre-B-cell proportions in the bone marrow during SLE in NZB/W mice (57). It is worth noting that treatment with HDAC6i upregulates alpha-tubulin acetylation and reduces the activity of cytoplasmic HDACs of B cells and restores the percentage of B lymphocytes (142). Based on the study that Hsp90 hyperacetylation has been proven to reduce Hsp90 function; hyperacetylated α-tubulin and Hsp90 of B cells were observed in mice depleted of HDAC6 and those treated with HDAC6 small interfering Ribonucleic Acid (siRNA) (144). All these findings indicate that HHDAC6 may have a critical role in the dysregulation of B-cell development during rejection, that restored HDAC6 expression contributes to immunopathogenesis, and that HDAC6i appears to be advantageous in the prevention of rejection through the acetylation of key signaling and transcription factors involved in the activation of the immune system. Altogether, the limited success of these reported strategies has provided novel insights on HDACi6 plasticity into improvement to prevent allograft rejection, which will eventually be beneficial in inducing the future development of immune tolerance in clinical trials.

### 4.3 Mechanism and histone deacetylase 6 of renal graft fibrosis

**Figure 4:**
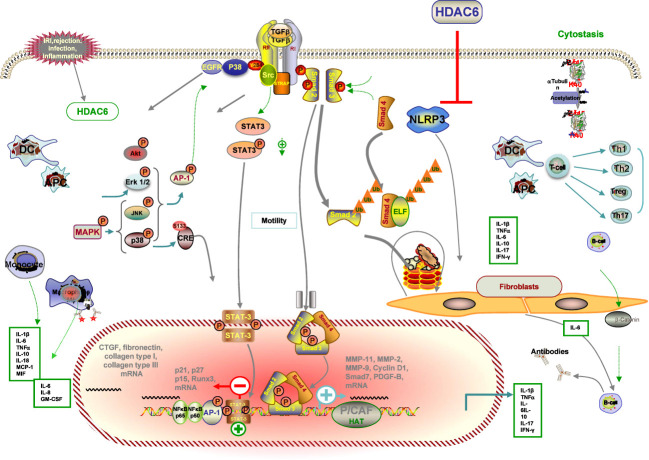
Schematic illustration of the anti-fibrotic property of HDAC6i in renal transplantation. Injured tissue or activated immune cells secrete profibrotic factors that induce fibroblast differentiation into myofibroblasts. Myofibroblasts actively synthesise extracellular matrix. HDAC6i negatively regulates fibrosis. (1) In the initial stage of the pro-fibrotic process, intra-graft inflammation, an integral part of the host defence mechanism in response to injury, is activated. Through modulation of p38 MAPK, NF-kB and AP-1 pathways, several pro-inflammatory, pro-fibrotic cytokines and adhesion molecules (MMP-1, MMP-2, MMP-9, MCP-1) are secreted by tubular cells with consequent recruitment of inflammatory infiltrates (lymphocytes, macrophages, DCs, neutrophils) that may facilitate the recruitment of new interstitial mononuclear cells. (2) Macrophages are a major source of TGF-b, which modulates the production of IL-1β, TNF-α, IFN-bγ and IL-10 and induces myofibroblast differentiation and the production of ECM proteins. (3) Damaged mitochondria mediate apoptosis and necrosis with the consequent release of DAMPs, which activate the NLRP3 inflammasome and participate in the prosis of fibrosis. (4) ROS accumulation via IRI, NADPH oxidase and mitochondrial dysfunction contribute to EMT. (5) Inhibition of HDAC6 attenuated EGFR, TGF-b1 induced EMT markers, which is accompanied by HDAC6-dependent deacetylation of a -tubulin. (6) The underlying mechanism by which HDAC6 inhibition can attenuate fibrosis by inhibiting profibrotic genes, multiple activations of cell pro-fibrotic signalling pathways and inflammation. Dashed arrows indicate indirect pro-fibrotic effects.

The progression and development of renal injury after RT ultimately contributes to renal interstitial fibrosis in chronic renal allograft injury, which is also the major precipitating factor in the decline of renal function (145). Several factors, including IRI, acute rejection, various types of infection, and chronic allograft dysfunction, can lead to inflammation in the recipient’s kidney, which is a major consequence of the development of chronic hypoxia, ultimately leading to renal fibrosis (6). Emerging evidence implicates HDAC6 in renal fibrogenesis (146) (**Figure 4**).

Accumulating studies from past and present have reported the significant role of HDAC6 implicated in inflammatory and fibrotic disorders in a model of inflammation in a deacetylase activity–dependent manner, which we also mentioned in the previous IRI part. A previous study demonstrated that genetic knockdown and pharmacological HDAC6i regulated inflammatory cytokine production in response to multiple pathogen stimuli such as LPS, *Clostridium difficile* Toxin A, and HIV-1 Tat. This supports previous findings that HDAC6 overexpression induces ROS generation and inflammatory cytokine secretion (147). It was previously shown that HDAC6i also attenuated LPS-induced macrophage activation and production of proinflammatory cytokines, including TNF-α, IL-1β, and IL-6, while reducing acetylated α-tubulin (148). In line with the changes in inflammatory cytokines, there was a significant attenuation of nicotine-induced macrophage pyroptosis by HDAC6 deficiency by TA or siRNA, confirmed by decreased protein expression levels of NLRP3, cleaved caspase1, IL-1β, IL-18, and LDH (149). In addition, administration of HDAC6i by TA effectively suppressed the production of MPO, TNF-α, and IL-6 in the cisplatin-induced AKI, as well as the prevention of damage to kidney function, together with the survival improvement in a rodent model of subsequent CLP (150). Given the known effect of regulating the expression of inflammatory cytokines, HDAC6 also modulates other proinflammatory factors such as monocyte chemoattractant protein-1 (MCP-1), which is thought to play an important role in monocyte recruitment during acute inflammation (75). Studies have also shown that monocyte/macrophage migration into the peritoneal cavity is reduced by the loss of HDAC6 (89).

It has been observed that TubA could reduce the increased expression and localization of connective tissue growth factor (CTGF), a well-known pro-fibrotic factor, in kidney tissue sections after ANG treatment (98). The mRNA and protein levels of fibrosis marker genes (fibronectin, collagen type I, and collagen type III) were significantly increased in renal fibrosis in hypertensive mice compared to the control sham group, which was blocked by TubA treatments or HDAC6 siRNA. Similarly, in response to ANG stimulation, immunohistochemistry showed that TubA administration alleviated FSP1 expression, a specific hallmark of renal fibrosis, in the interstitial region (52). Emerging research has shown that collagen type 1 is a novel substrate for acetylation and deacetylation by p300 and HDAC6, respectively. Moreover, knockdown of the p300 transcriptional coactivator reduced collagen type I acetylation, in contrast to TubA, which ultimately modulates collagen expression (52).

A wealth of experimental evidence supports the diverse role of HDAC6 in the regulation of key transcription factors of inflammatory signaling (98). A previous finding indicated that HDAC6 modulates inflammatory expression, which may result from modulation of the p38 MAPK, NF-κB and AP-1 pathways, or perhaps other underlying pathways (99). Activation of the NF-κB pathway, a canonical inflammatory pathway, is engaged in the development of AKI and is responsible for the transcriptional induction of a variety of proinflammatory cytokines and a number of chemokines (98). Studies in mouse models of inflammation have implicated the overexpression of HDAC6 in the abnormal activation of inflammatory signaling pathways, including the NF-κB pathway, in cisplatin-induced AKI (53, 95). The level of NF-κB p65 phosphorylation was significantly promoted in the cytoplasm and reduced in the nucleus by pharmaceutical HDAC6i with TA (99). Confocal microscopy showed that TA apparently blocked nicotine-induced p65 nuclear translocation (151). In agreement with previous reports, it has also been shown that HDAC6 inhibition by TA pretreatment restores site-specific acetylation of p65 and reduces its nuclear translocation in another model. In addition, p65 has been implicated in NLRP3 transcription and macrophage pyroptosis (151). Taken together, it can be concluded that HDAC6 inhibition by TA attenuates cisplatin-induced inflammatory cytokine expression in AKI and nicotine-induced macrophage pyroptosis by reducing NF-κB phosphorylation (151, 152).

Increasingly, previous research has indicated that persistently high expression of the phosphorylated epidermal growth factor receptor (EGFR) signaling pathway is also involved in the fibrotic kidney, suggesting that the EGFR signaling pathway also plays a key role in mediating angiotensin II–induced renal fibrosis. ACY-1215 treatment also attenuates renal fibrosis by inhibiting Unilateral Ureteral Obstruction (UUO)-induced phosphorylation of EGFR and its downstream signaling protein molecules (98). TGF-β, the most important marker of interstitial fibrosis, is of prime importance in synthesis and degradation of epithelial–mesenchymal transition (EMT) by inducing transcription of collagen genes and inducing EMT of tubular epithelial cells, leading to interstitial fibrosis and chronic allograft dysfunction (153). In addition, from another work on idiopathic pulmonary fibrosis, it is known that inhibition of HDAC6 attenuated TGF-β-induced EMT markers released from the complex by macrophages and activated by inflammation, accompanied by HDAC6-dependent deacetylation of α-tubulin (154). Mechanistically, HDAC6 is shown to influence epigenetic histone modification, leading to renal fibrosis and TGF-β1-dependent nuclear accumulation of Smad3-dependent fibrotic genes, and inhibition of HDAC6 attenuated renal fibrosis and inflammation (58).

Collectively, selective HDAC6i can be involved in attenuating fibrosis by inhibiting profibrotic genes, multiple activations of cell pro-fibrotic signaling pathways, and inflammation. The favorable safety profile of HDAC6i and works presenting HDAC6 in multiple inflammatory and fibrotic diseases make sure the pathological change of selective HDAC inhibitors in the therapeutic strategy of RT-related fibrosis.

## 5 Limitations and future perspectives

With the elucidated mechanism of RT-related complications, HDAC6 has attracted a great deal of attention as a potential drug target. As the mechanism of RT-related complications induced by immune and non-immune injury has been elucidated, HDAC6 has attracted wide attention as a potential drug target. Due to its unique cytoplasmic localization, HDAC6 isoform regulates acetylation status of α-tubulin, HSP90, TGF-β, and peroxiredoxins. As an emerging class of drugs, selective HDAC6 inhibitors can act on many pathological links of various immune-related and renal diseases, such as oxidative stress injury, inflammatory response, apoptosis, B-cell and T-cell activation, Th cell differentiation, and macrophage responses, and have been shown to be beneficial in preventing disease progression. However, existing HDAC6 inhibitors also have some shortcomings, such as tubacin, which is useful as a research tool but is not suitable for clinical treatment due to its highly lipophilic properties, rapid metabolism in the body, and cumbersome synthesis (155). Although many contemporary HDAC6 inhibitors are efficacious in the immune system, only the DD2 domain has been well targeted in all small-molecule HDAC6 inhibitors, leading to off-target effects in pharmacological inhibitors due to limited selectivity (156). The absence of another ubiquitin-binding zinc-finger domain could result in discrepancies between experimental observations made with pharmacological inhibitors and genetic knockout approaches, the latter targeting both catalytic domains. It will be interesting to explore whether other targets contribute to HDAC6’s role in inflammation and inflammatory disorders. Additionally, some HDAC6 inhibitors do not display high selectivity and may inhibit related HDACs, and the newly discovered HDAC6 inhibitors are still in the experimental stage (157, 158). Additionally, it should be mentioned that although there is limited literature on RT directly, the conclusions in other organ transplantations, fibrosis and IRI models can strongly prove the effectiveness and feasibility of HDAC6 application in the field of RT. Thus, further basic research in preclinical models is necessary for the identification of novel high-potency HDAC6 inhibitors with superior selectivity. Finally, regarding its role in immune responsivity, HADC6 may be involved in the recurrence of primary kidney diseases in RT; however, additional studies are needed to confirm this hypothesis.

## 6 Conclusion

A substantial amount of validated studies has identified HDAC6 as a pivotal modulator of inflammation and a promising therapeutic candidate for the management of a variety of renal diseases. Even more significantly, small-molecule inhibitors of HDAC6 have not been accompanied by severe toxic effects. The highly selective HDAC6 inhibitor TA has been evaluated in the therapeutic areas of neurodegenerative diseases, cancer, and COPD with no apparent adverse effects. Over the past few decades, HDAC6 has been identified as a relatively attractive target for the development of drugs to treat a wide range of diseases. These studies have significantly improved our understanding of HDAC6 function in the kidney and support the potential evaluation of HDAC inhibitors in kidney transplant patients. In view of the innovation of these inhibitors, more work is needed to substantiate their role as a promising intervention and to address unknowns before clinical translation.

Collectively, the favorable profile of HDAC6 inhibitors and the identification of HDAC6 in a series of inflammatory disorders offer compelling opportunities for the advancement of selective HDAC inhibitors in the management of RT-associated complications. Important evidence on the HDAC6 apparatus particularly involved in the pathogenesis of renal complications is encapsulated in the present review through modulation of innate and adaptive immune responses, which focuses on therapeutically relevant avenues with considerable translational promise to the clinic.

## Author contributions

Q-qZ and SC mainly contributed to the conception, the design, and the writing of the manuscript. Q-qZ wrote, prepared, reviewed, and edited the manuscript; W-jZ reviewed, revised, and commented on this article and funding acquisition; Q-qZ and SC supported the final draft editing and revised the manuscript critically for final acceptance for publication. SC supported and supervised the overall design of the article. Q-qZ conceived of all figures. All authors contributed to the article and approved the submitted version.
